# The Purple Tomato Story; From Laboratory Bench to the Consumer

**DOI:** 10.1021/acsfoodscitech.4c00692

**Published:** 2024-11-05

**Authors:** Cathie Martin, Eugenio Butelli

**Affiliations:** John Innes Centre, Norwich Research Park, Norwich NR4 7UH, U.K.

**Keywords:** anthocyanin, polyphenol-enriched, tomatoes

## Abstract

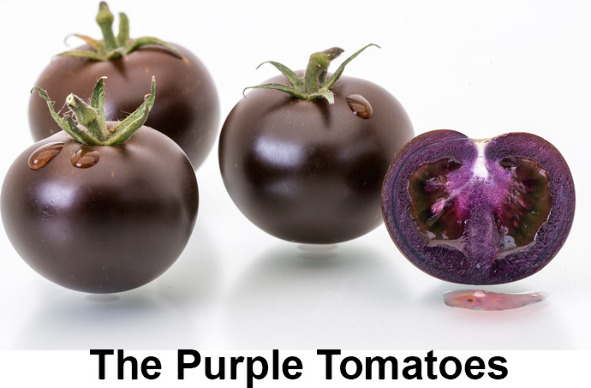

A world apart from academic research, the path from developing a polyphenol-rich crop to a product available to consumers is not one taken by many research scientists. Here we review the steps taken to commercialize anthocyanin-enriched purple tomatoes in the USA. In describing some of the difficulties encountered and the work that was necessary for a successful commercial launch of a new biotech product, we hope to encourage others to believe that there is a viable route to market, and an appetite for polyphenol-enriched foods that can protect health.

## Introduction

One of the most pressing challenges for the next 50 years is to reduce the impact of chronic disease including cardiovascular disease, diseases related to the Metabolic Syndrome, certain cancers, and age-related degenerative diseases. The risk of chronic disease is strongly associated with three socio-behavioral risk factors; smoking, lack of physical activity, and unhealthy diet.

The problem of unhealthy foods has come to be associated with the “bad” things they contain; trans-fats, sugars, salt, flavor enhancers, and other additives. However, a major problem with ultraprocessed/junk foods is that they lack many “good” things including vitamins, fiber, and important phytonutrients, often classified as “antioxidants”. While the essential roles that vitamins play in the human diet and their importance in avoidance of nutritional diseases have long been known, appreciation of the significant role that phytonutrients play in health promotion and disease prevention is much more recent. Part of the rise in chronic disease resulting from unhealthy eating, especially diseases associated with obesity and Metabolic Syndrome, is due to the increased consumption of diets poor or deficient in phytonutrients. There is good epidemiological evidence to support the idea that increased consumption of diets rich in phytonutrients derived from fruit and vegetables can maintain health, enhance life expectancy, and improve the quality of life of all individuals.

Polyphenols are a group of phytonutrients made by all land plants and used widely for their own defense against biotic and abiotic challenges. While polyphenol phytonutrients have been actively researched for their ability to promote health for more than 20 years, most attention has been focused on selected polyphenols that can be obtained from a limited range of foods such as epigallocatechin gallate (EGCG) from green tea, genistein from soya, curcumin from curry spice and resveratrol from red wine.^1^ For these polyphenols, there are preclinical data as well as a wealth of studies, *in vitro*, confirming that they confer cardioprotection and have cancer chemopreventive effects for at least some consumers. Nothing like this amount of information is available for polyphenol phytonutrients which are much more widely available in the diet, such as anthocyanins, perhaps because the opportunities for intellectual property (IP) and product protection of these phytonutrients are much more limited, detracting from commercial sponsorship of preclinical and large-scale intervention studies. The outputs of several EU-projects have been exciting, with data suggesting that anthocyanins, the plant pigments available in a range of fruits (particularly berries) and some vegetables, also offer significant cardioprotection and delay the progression of cancer in preclinical studies.^2−7^ Anthocyanins are pigments produced by most higher plants, and as part of the human diet, anthocyanins offer protection against a broad range of human diseases. Anthocyanins are widely available in traditional diets such as the “Mediterranean” diet and may be one of the active principles underpinning why this type of diet, consisting mostly of grains, fruits, beans, and vegetables, may help to prolong life, lower the risk of heart disease and cancer, and lower blood cholesterol levels.

**Figure 1:**
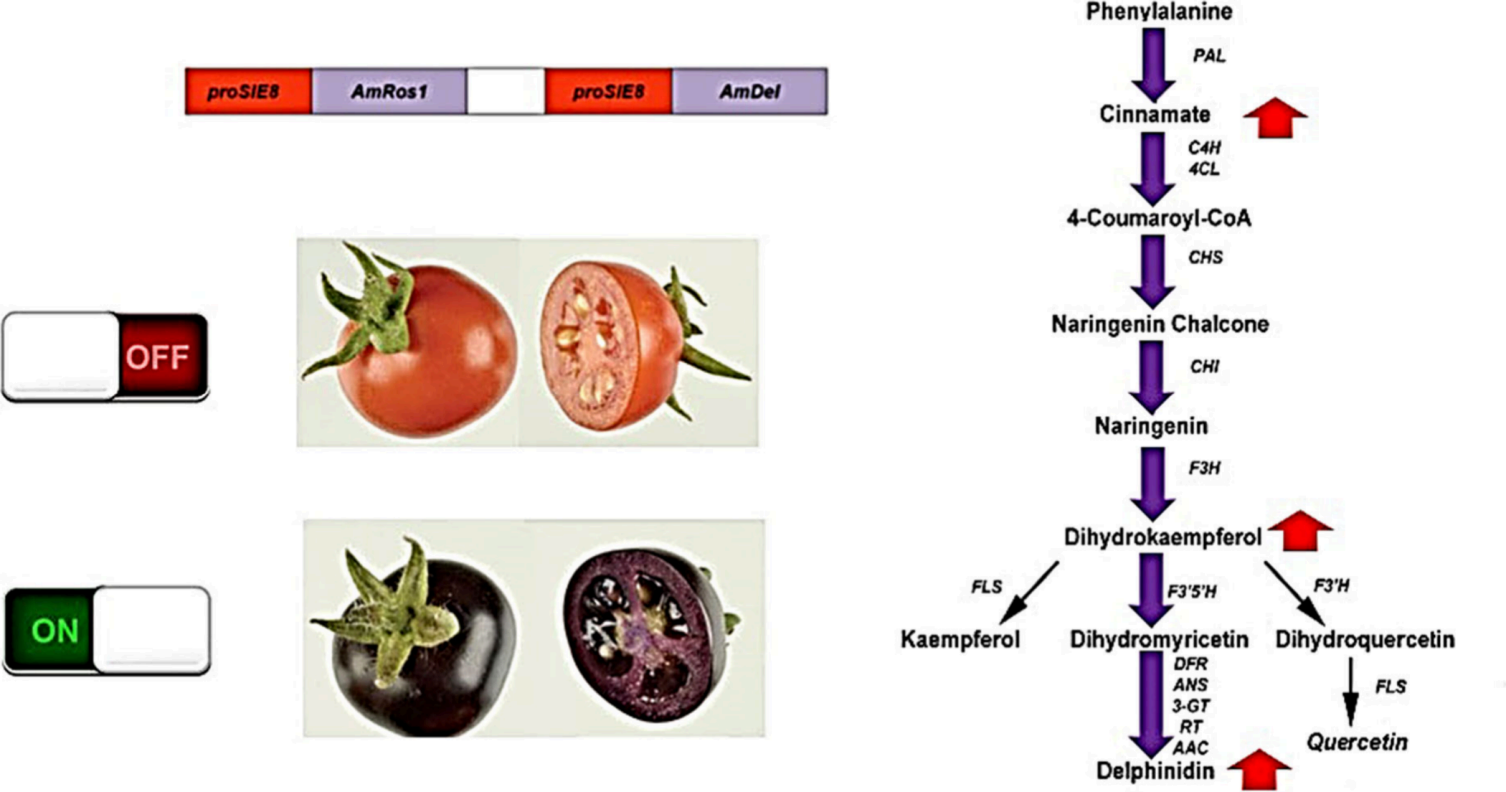
The objective for creating purple tomatoes. In normal red tomatoes, the transcriptional complex that regulates anthocyanin biosynthesis is switched off; no anthocyanins are made in fruit which are red due to their high content of the carotenoid, lycopene. Introduction of two of transcription factors (Rosea1 and Delila) with expression specifically in fruit causes induction of anthocyanin biosynthesis (particularly delphinidin) and the development of purple fruit. Health benefits can be assessed by comparing health status of disease models on diets containing red and purple isogenic foods.

Between 2002 and 2005 we collaborated with a team of scientists from Europe on the EU Framework 5 consortium program, PROFOOD which aimed to increase the content of flavonoids in plant species like tomato. We identified transcription factors (called Rosea1 and Delila) that modulated anthocyanin metabolism and established the specificity of these transcription factors in terms of their target genes. We built gene constructs for modulated expression of these transcription factors to increase the levels of phenylpropanoid antioxidants in tomato fruits. (Figure 1)

**Figure 2:**
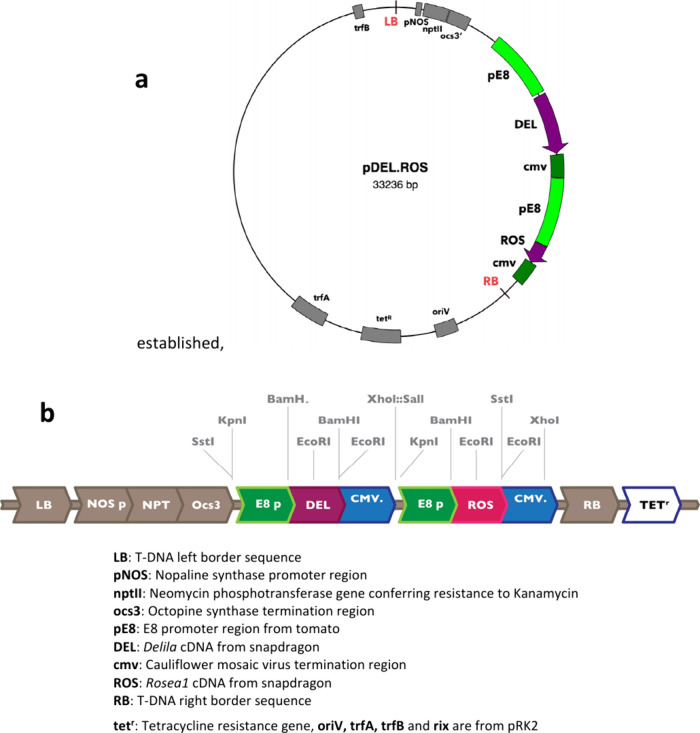
Schematic representation of binary vector pDEL.ROS used to produce purple tomatoes. a) Construction of pDEL.ROS in vector pRK290 which carries a tetracyclin resistance gene (tet) for selection in bacteria. b) Detail of gene arrangements in pDEL.ROS including promoters (NOSp and E8p) 3′sequences (Ocs 3, CMV), genes encoding transcription factors (*DEL, ROS*) and *npt*II (NPT) gene encoding kanamycin resistance. Plasmid construction: The 2175bp *E8* promoter was amplified from the tomato genomic DNA by PCR. The promoter was cloned in pJIT60 to replace the CaMV 35S promoter and in pJAM1500, (pJIT60 containing a Gateway Destination Cassette; Invitrogen) such that *E8* replaced the *35S* promoter. This resulted in plasmids pE8.60 and pE8.1500 respectively. The region containing E8-Gateway-CaMV 3′ sequence from pE8.1500 was cloned in pSLJ7291 resulting in plasmid pSLJ.E8.1500. The full-length *Delila* cDNA was amplified by PCR and inserted in this plasmid using Gateway recombination technology, resulting in the binary construct pSLJ.E8.DEL. The full-length *Rosea1* cDNA was amplified by PCR and inserted in the plasmid pE8.60, resulting in plasmid pE8.ROS. After the introduction of a double stranded oligonucleotide containing a *Sal*I restriction site in this plasmid, the region containing E8-*Rosea1* cDNA-CaMV3′ sequence was cloned as a *Sal*I*-XhoI* fragment into the *XhoI* site of pSLJ.E8.DEL resulting in the binary construct pDEL.ROS.

In any attempt to improve crops through metabolic engineering, the amounts of target metabolites induced are of primary importance. For applications, changes in flux need to be large, meaning that much of the metabolic engineering that has been reported for crop plants has not yet been applied successfully. With the objective of producing fruit with high levels of anthocyanins, the two transcription factors were expressed in tomato fruit, resulting in fruit which displayed an intense purple coloration in both peel and flesh (Figures 1 & 2). We initially measured anthocyanins in transgenic fruit, and we observed total anthocyanin levels up to 2 mg/g fresh weight dependent on the line analyzed. Two major anthocyanins were identified delphinidin–3-*O*-(*p*-coumaroyl)-rhamnose-glucoside, 5-O-glucoside and petunidin-3-O-(*p*-coumaroyl)-rhamnose-glucoside, 5-O-glucoside in the lines expressing both *Rosea* and *Delila* in fruit. These anthocyanins were not detected in the control fruit.

We measured the total antioxidant activity of wild type and transgenic fruits and showed that the antioxidant activity was directly correlated to the amount of anthocyanin accumulated and was increased up to 2.8-fold in transgenic fruits expressing both *Rosea* and *Delila* compared to wild type fruit.

The ability of dietary anthocyanins to limit the progression of cancer was tested by preparing rodent pellets supplemented with 10% (w/w) powder from freeze-dried tomatoes, either red control tomatoes or the purple high-anthocyanin tomatoes. These diets had been fed to control mice and shown to have no effect on the food consumption, growth, development, or body weight gain compared to the standard diet. Three diets were fed to p53 knockout mice, which are highly tumorigenic and provide a reproducible whole animal model for cancer. p53 knock out mice had an average life span of 140 days on the standard diet, and no difference in average life-span was observed in mice on the red tomato-supplemented diet. However, p53 knockout mice fed the purple tomato supplemented diet showed an average life span of 183 days, an increase of 30% compared to the standard diet. This work was published in Nature Biotechnology^3^ and established, for the first time using isogenic foods, a beneficial effect of dietary anthocyanins on cancer progression and mortality in preclinical studies.

The public interest that followed the publication of this work was almost overwhelming. Despite recommending that consumers could get similar benefits from consumption of berries rich in anthocyanins such as blackberries and blueberries, the public demand for purple tomatoes was high, as gauged from scientific presentations, newspaper and international radio and TV interviews, and public and stakeholder engagement fora, perhaps because people thought that results from anthocyanins in one food might not translate effectively to those from other foods. Indeed, there are very few studies that have examined whether the beneficial effects of one specific anthocyanin are shared by all anthocyanins equally or whether anthocyanins in different plant foods have differential health benefits when included in the diet. However, because the tomatoes were “genetically modified”, we could find no companies in the UK, Europe, or even in the USA with any interest in securing a route to market. For any product improved by scientific design, translation requires production of a commodity available to the general public. Commercialisation is essential, and in response to the clear public interest in the high anthocyanin, purple tomatoes, we formed a spin–out company, which we named Norfolk Plant Sciences (NPS) from the John Innes Centre and the Sainsbury Laboratory in Norwich, Norfolk, UK.

The aim of NPS was and is to find ways of commercializing research into plants with increased levels of health-giving compounds such as antioxidants. This review will review the steps we took for regulatory approval of purple, high anthocyanin tomatoes in the USA and hopefully provide a template for initiatives started by others to use plant biotechnology for crop improvement.

## Development of a Business Plan

A standard business plan for a spin-out would include filing IP and appointing a business development manager to devise a business plan. The aim would be to seek funding from angel investors and/or to license the IP to larger commercial organizations interested in taking the business forward. None of these options were particularly well suited to commercialization of purple tomatoes. First, the work was published in November 2008 just before the global financial crash, which made investors even more reticent than usual about investing in new enterprises. Second the regulatory landscape for GMO crops was extremely challenging, with the European Union, of which the UK was then part, exercising an effective moratorium on new approvals and even in the USA “deregulation” (meaning that the product had been approved by USDA/APHIS for cultivation and commercialization and no longer required regulatory oversight) reputedly costing over $100 million for each new biotech crop. Given the timelines and projected costs required for “deregulation” in the USA, angel or venture capital investment eluded NPS and was realistically unfeasible because our product did not have adequate value, and the timelines available with such support were much too short for products developed using horticultural biotechnology. The realization that the normal route was not a viable option for NPS did not come immediately. We learned by trying to raise money from such investors only to be eventually rebuffed by every single organization we approached. This also meant that we ran through a significant number of CEOs, each with a standard business plan but none of whom got us any closer to a commercial product.

We did make progress on IP by filing a broad ranging patent that was subsequently divided into two, both of which were granted in the USA, giving NPS freedom to operate in engineering flavonoid production in Solanaceous crops. These two patents served as vital assets for the company in all subsequent business interactions. Our second achievement was to appoint a proactive scientific advisory board, members of which had expertise in agricultural and horticultural biotechnology and all of whom were sympathetic to the idea that we had a product attractive to consumers that could change attitudes to GMOs for the better. The third development was to abandon the position of CEO and to appoint a local businessman as Chairman of the NPS Board of Directors, who put our accounts in order and encouraged the company to raise relatively small amounts of money through engagement in academic collaborations, where company expertise could contribute to the advancement of other horticultural biotechnology products and initiatives.

Despite our initial optimizm, in 2012 the NPS scientific advisory board held an emergency meeting to discuss how to move the company forward. Remarkably, far from encouraging us to fold the company because of lack of interest or investment, the outcome of that meeting was the suggestion that there might be a way to get a “purple tomato product” onto the market in the USA without the cost and time investment required for “deregulation”. This idea focused on producing tomato juice as a product that would not present any perceived risk to the environment and which could be grown in greenhouses, to avoid the approvals required for “deregulated cultivation”. We were advised that a number of GMO biotech products were already being developed in the USA based on this model and that requesting that the FDA consider whether the GMO purple tomato product was safe for human consumption would be important for supporting the product. We proceeded to work on this revised plan for commercialization by engaging with FDA about what they needed for successful “notification”.

## Voluntary Premarket Consultation with FDA

We began our discussions with the FDA in 2014 and completed our application for voluntary notification at the beginning of March 2020. FDA informed us that when assessing the safety of purple tomatoes for human food use, they would note that the intended human food uses were the same as for other tomatoes on the US market, that tomato is consumed fresh, in salads, as well as a processed food, and that the purple tomato was not intended for use in animal food in the United States.

Preparing an application for voluntary consultation by the FDA was not straightforward. Although termed a Biotechnology Notification Form (BNF), there was no actual BNF available to complete. We assume that the absence of a form allows FDA greater freedom and flexibility over the inquiries they can make. Instead, we had to submit an application to the FDA under the Freedom of Information Act to receive a copy of a recent successful application for voluntary consultation. This provided a rough idea of the types of information required, along with the necessary level of detail.

We compiled a dossier of information on the selected purple tomato line (event), which addressed the questions FDA needed to assess safety for human consumption. This dossier provided information on the selected purple tomato event on which the FDA could base their assessment of the safety of the fruit as a food for human consumption.

### 1 Transformation Process and Vector Construction

Tomato *(Solanum lycopersicum* cv MicroTom) plants containing the selected purple tomato transformation event were produced at the John Innes Centre (Norwich). The transformation procedures and results of this work were reported in detail in Nature Biotechnology by Butelli et al. in 2008.^3^ The line (event) taken forward for commercialization contained a single T-DNA insertion on Chromosome 4 of tomato, referred to hereafter as “the purple tomato event”.

### 2 Transgene Constructs in the Genetically Modified Plants

The number of T-DNA copies and number of loci were estimated using q-PCR analysis of progeny obtained by the self-pollination of T0 plants. This analysis indicated that T-DNAs had integrated at two distinct Mendelian loci, each harboring one T-DNA copy. For one locus, T-DNA insertion occurred on chromosome 4 at position 62904771. For the second locus, flanking sequences were retrieved but corresponded to an unanchored genomic contig (insertion at position 14105). However, this insertion was lost by segregation in subsequent generations and was not considered further in the characterization of the purple tomato event. The single T-DNA insert on Chromosome 4 (locus B) did not interrupt any existing open reading frame (ORF) or create new functional ORFs. In addition, PCR analysis using a range of nested primers confirmed the absence of the tetR gene in the genomes of transgenic plants containing the purple tomato event.

**Figure 3:**
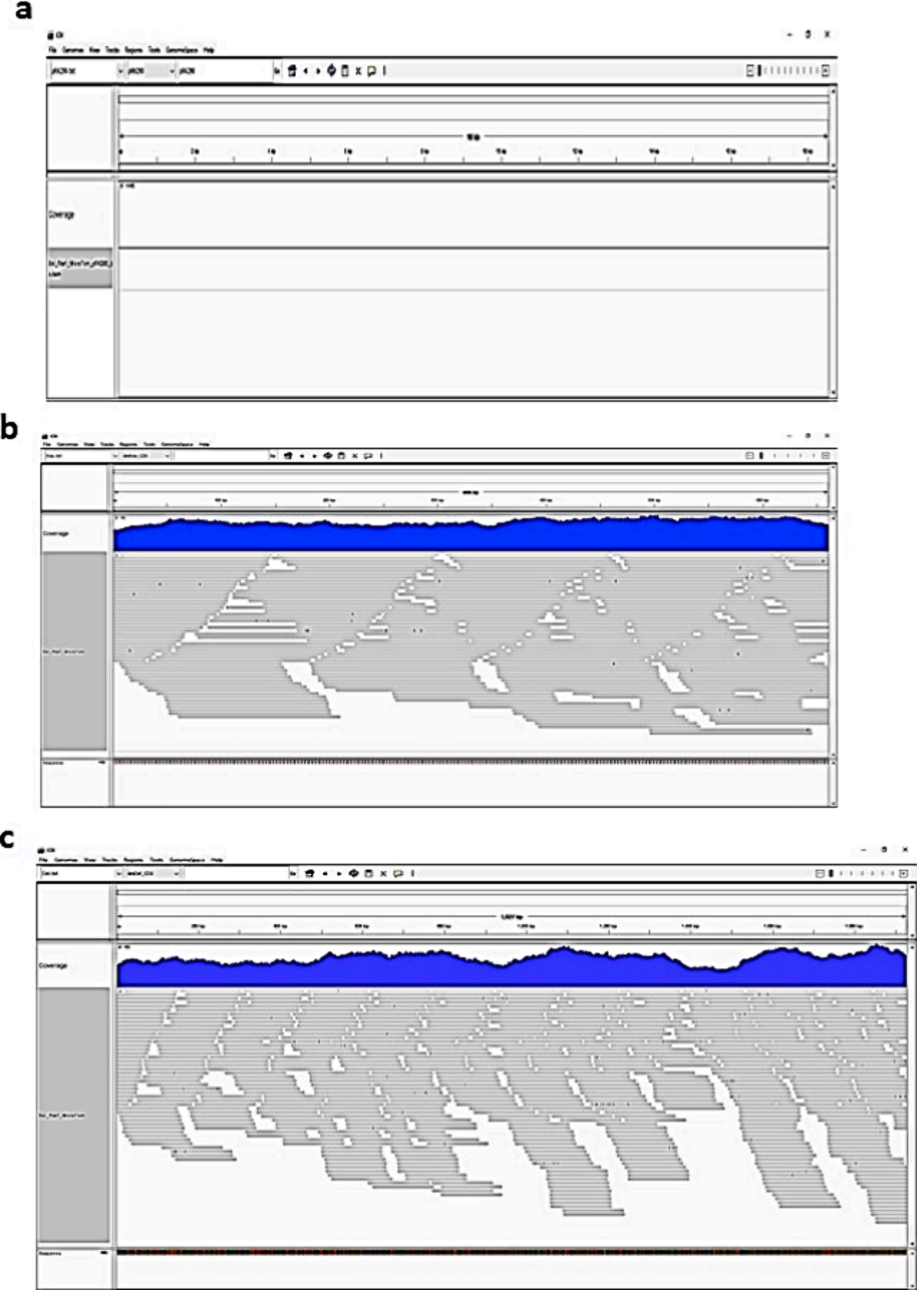
Analysis of genome sequence of tomato plant carrying the purple tomato transformation event (a) Results of search of purple tomato genome sequence for sequences of 150 nucleotides or greater with identity to pRK290 sequence taken from CLD04541: nucleotides 5854–25385 deposited in NCBI as AF184978.1. (https://www.ncbi.nlm.nih.gov/nuccore/AF184978.1). The purple tomato genome sequence has been deposited in ENA accession number ERR3500875. No reads aligned to this sequence with 100% identity over 150 nucleotides as shown in the alignment deposited in ENA under accession number ERR3502329. (b) Alignment of reads from Illumina hi-seq over the sequence of the *Rosea1* (*Ros1*) gene in the sequenced purple tomato plant. Aligned reads are shown as gray bars. Dark lines within gray bars show sequence mis-matches to the sequence of pDEL.ROS in individual reads. The dark blue profile shows the degree of read coverage for each part of the gene sequence. This alignment has been deposited in ENA as accession number ERR3502327. (c) Alignment of reads from Illumina hi-seq over the sequence of the *Delila* (*Del*) gene in the sequenced purple tomato plant. Aligned reads shown as gray bars. Dark lines within gray bars show sequence mis-matches to the sequence of pDEL.ROS in individual reads. The dark blue profile shows the degree of read coverage for each part of the gene sequence. This alignment has been deposited in ENA under accession number ERR3502327.

The complete genome sequence was determined for the purple tomato event in progeny of the T6 generation. No alterations in the introduced transgenes were detected and no vector backbone sequences were identified in the sequence from reads constituting >50-fold coverage (Figure 3). Use of genome resequencing to confirm a lack of insertion of vector DNA and the integrity of the transgenes proved to be a very effective and economical way of establishing these parameters important for food safety.

### 3 Inheritance and Stability of Inserted DNA

The transgenes at insert B were inherited stably over more than 6 generations and could be transferred to other genetic backgrounds with no loss of phenotype (Moneymaker, Ailsa Craig and VF36) by cross-pollination.^3^ The purple phenotype was introgressed into an industrial processing tomato variety (Ohio 8243) for the large-scale production of purple tomato juice.

### 4 Choice of Comparator and Production of Material for the Compositional Assessment

For compositional analyses, tomato cultivar “Moneymaker” containing the selected purple tomato event was compared with the same wild-type (wt) cultivar (Moneymaker). For the analysis of purple tomato juice, the product of fruit from the purple tomato event that introgressed (F4) into a processing variety of tomato (Ohio 8423) was compared to the product of fruit from Ohio 8423.

### 5 Anthocyanin Compositional Analysis

**Figure 4:**
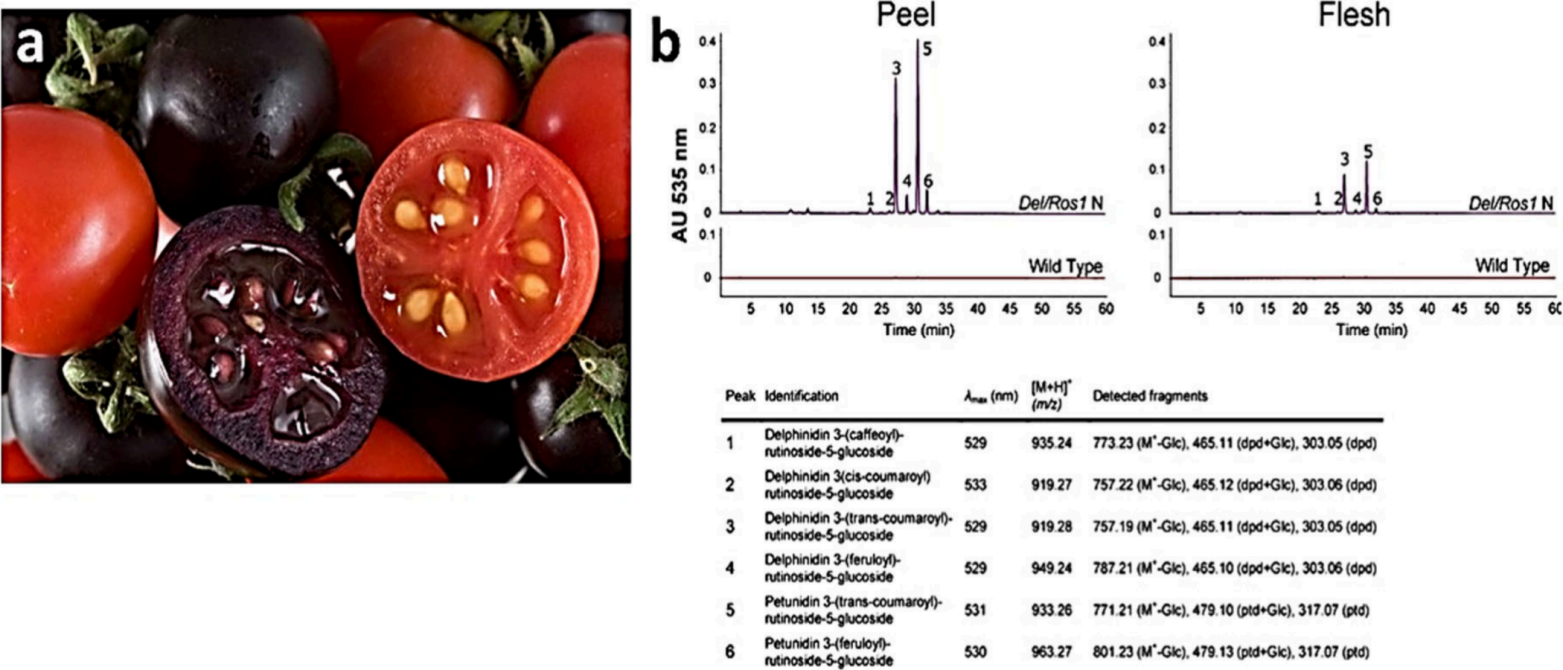
Fruit-specific phenotypes of purple tomatoes expressing both Del and Ros1 under the control of E8 promoter. (a) Purple and wild-type (red) tomatoes (b) Comparative analysis of phenylpropanoid content and composition in purple vs wild type tomato fruit. HPLC chromatogram of methanol extracts from the purple line and wild- type (red line) tomato fruit. HPLC analysis, recorded at 535 nm of extracts from peel or flesh of ripe fruit. Peaks marked with numbers represent anthocyanins. Classification and identification of methanol soluble compounds was performed based on PDA absorbance and ESI-Q-TOF mass spectrometry. The purified compounds were analyzed by HPLC and ESI-MS/MS. Spectral characteristics, molecular ions and fragments obtained are tabulated. Identification was confirmed by hydrolysis and HPLC analysis of the respective acyl and sugar moieties.

The alterations of gene expression and the analysis of total anthocyanin content in purple tomato fruits were reported in Nature Biotechnology^3^ (Figure 4). The fruit-specific expression of Delila and Rosea1 transcription factors increased the transcript levels of almost all of the genes encoding enzymes required for anthocyanin biosynthesis, genes encoding enzymes required for side-chain modification such as a putative anthocyanin acyltransferase, and two genes likely involved in the transport of anthocyanins into the vacuole. Anthocyanins were virtually undetectable in wild-type fruit but averaged 2.83 ± 0.46 mg of anthocyanin per gram of fresh weight in hemizygous “Micro Tom” plants containing the purple tomato event. High levels of anthocyanins were detected using high-performance liquid chromatography (HPLC) in both the peel and flesh of purple fruit, the major ones being anthocyanin 3,5-diglucosides acylated with coumaric acids. There were no increases in flavonols in the flesh of purple tomatoes; however, the main natural flavonol, rutin, was detected in the peel of purple fruit, while being barely detectable in the peel of wild-type tomatoes. Additional analyses showed that the contents of the major phenylpropanoids (chlorogenic acid, flavonols and anthocyanins) were increased significantly in the purple tomato fruits compared to wild-type.^8^

### 6 Compositional Analysis

Compositional analysis was carried out by Eurofins (Eurofins Food Testing UK Ltd., (http://www.eurofins.co.uk) to ensure that analysis met GLP accredited standards. Whole, fresh, ripe tomatoes from the cultivar Moneymaker containing the purple tomato event were compared with wild type (WT) Moneymaker tomatoes. Compositional analyses of the purple tomatoes provided levels of proximates in line with levels found in the literature; changes in alkaloid levels remained small. (Table 1). There were <25% differences in proximates (glucose, fructose, total carbohydrate (CHO), protein, total fat and fiber), vitamin C, the minerals Mg, K and Na, total lycopene and β-carotene between the purple Moneymaker and the Moneymaker wild-type. There were >25% differences in folate and α-tomatine between the purple Moneymaker and the Moneymaker wild-type. However, all contents were within the ranges reported in the scientific literature for commercial tomato varieties.^9^ α-Tomatine concentrations were 3.36-fold higher in the purple Moneymaker compared to Moneymaker wild-type tomatoes which was in keeping with the slower ripening rate of the purple Moneymaker tomatoes that was reported by Zhang et al., (2013).^8^ All α-tomatine concentrations were at the low end of the range of concentrations reported for multiple tomato varieties in the scientific literature.

**Table 1:** Nutrient Composition of Tomatoes–Standard, Raw

**Analyte**	**units**	**RED**	**PURPLE**	**USDA (avg)**	**USDA (min)**^a^	**USDA (max)**	**McCance and Widdowson 2019**^b^
*moisture*	g/100g	94.72 ± 0.12	95.1 ± 0.12	94.52	92.7	95.73	94.6
*Crude protein*	g/100g	0.64 ± 0.05	0.70 ± 0.09	0.88	0.59	1.06	0.5
*Ash*	g/100g	0.32 ± 0.02	0.48 ± 0.02	0.50	0.37	0.60	n/a
*CHO*	g/100g	3.06 ± 0.29	3.26 ± 0.27	3.89	n/a	n/a	3
*Fructose*	g/100g	1.46 ± 0.05	1.22 ± 0.09	1.37	1.1	2.32	1.6
*Galactose*	g/100g	<0.1 ± 0	0.1 ± 0	0.00	0.00	0.00	0.00
*Glucose*	g/100g	1.26 ± 0.02	1.04 ± 0.08	1.25	0.49	2.67	1.4
*Lactose*	g/100g	<0.1 ± 0	0.1 ± 0	0.00	0.00	0.00	0.00
*Maltose*	g/100g	<0.1 ± 0	0.1 ± 0	0.00	0.00	0.00	0.00
*Sucrose*	g/100g	<0.1 ± 0	0.1 ± 0	0.00	0.00	0.02	0.00
*Total sugar*	g/100g	2.72 ± 0.06	2.24 ± 0.17	2.63	1.59	5.01	3.00
*Total fiber AOAC*	g/100g	1.1 ± 0.2	0.7 ± 0.1	1.2	0.7	2	1
*Energy kcal*	kcal/100g	18.4 ± 0.83	16.8 ± 0.77	18	n/a	n/a	4
*Energy kJ*	kJ/100g	81.8 ± 1.78	71 ± 2.95	74	n/a	n/a	61
*Total fat*	g/100g	<0.3 ± 0.04	<0.3 ± 0	0.2	0.07	0.80	0.10
*Salt*	g/100g	<0.025 ± 0	<0.025 ± 0	n/a	n/a	n/a	n/a
*Mono unsat FAs*	g/100g	<0.1 ± 0	<0.1 ± 0	0.031	n/a	n/a	0.03
*Poly unsat FAs*	g/100g	<0.1 ± 0	<0.1 ± 0	0.083	n/a	n/a	0.05
*Sat Fas*	g/100g	<0.1 ± 0	<0.1 ± 0	0.028	n/a	n/a	0.03
*Trans FAs*	g/100g	<0.1 ± 0	<0.1 ± 0	n/a	n/a	n/a	0
*Mg*	g/100g	0.01 ± 0.00	0.01 ± 0.00	0.011	0.007	0.015	0.008
*K*	g/100g	0.16 ± 0.00	0.23 ± 0.01	0.237	0.144	0.385	0.0223
*Na*	g/100g	<0.01 ± 0	0.01 ± 0.00	0.005	0.001	0.024	0.002
*beta carotene*	ug/100g	451.6 ± 42.4	661.8 ± 64.8	449	184	572	349
*Folate B9*	ug/100g	8.92 ± 0.28	14.22 ± 0.31	13.7	7.8	19.8	23
*Ascorbate C*	mg/100g	6.86 ± 0.18	8.1 ± 0.86	15	1	36	22
*Phylloquinone K1*	ug/100g	2.69 ± 0.14	1.99 ± 0.1	7.9	2.2	60	6
*Lycopene*	mg/kg	n/a	20.9	25.73	11.36	34.19	n/a

a Average and min/max values available online: USDA Food Composition Databases Show Foods—Tomatoes, Red, Ripe, Raw, Year Round, https://ndb.nal.usda.gov/ndb/, accessed 09–18–2018.

b McCance and Widdowson: The Composition of Foods Integrated Dataset 2019 available online: https://www.gov.uk/government/publications/composition-of-foods-integrated-dataset-cofid. From 22 samples of standard raw commercial tomatoes, accessed 08–25–2019.

### 7 Purple Tomato Phenotype

Tomato plants containing the selected transformation event exhibited a characteristic purple fruit phenotype that was stably inherited in the cultivar “Micro Tom”. This trait was also introgressed in other tomato varieties such as “Moneymaker”,^8^ “Ailsa Craig”, VF36 and into an industrial processing tomato variety (Ohio 8324) for the large-scale production of purple tomato juice. A comparable purple tomato phenotype can be obtained through classical breeding, such as in the commercial variety “Indigo Rose” from the Oregon State University, USA.^10^ However, in the latter case, the overproduction of anthocyanin leading to purple coloration is limited to the peel of the fruit. As expected, purple tomatoes differed principally from the comparator wild type MoneyMaker by their high content of anthocyanins. These same anthocyanins are found in significant amounts in Asian eggplant and purple heirloom potatoes. The levels of anthocyanins in the purple tomatoes are similar to those in blueberries and cranberries, but less than those in New Zealand black currants.^11^

Other metabolites differ slightly between purple tomatoes and comparators but levels in purple tomatoes fall within the ranges reported for commercial tomatoes.^9,12^

### 8 Toxicology, Allergenicity and Nutritional Assessment

Neither Delila nor Rosea1 proteins, produced in the purple tomato event, exhibited any significant sequence homology to known allergens, and we found that both were degraded rapidly in standard digestion tests. In addition, neither of these proteins could be detected in juice prepared from purple tomatoes, using ultrasensitive proteomic analysis by LC-MS.

Our estimates of exposure to anthocyanins from purple tomatoes are of the same order as those from consumption of other colored fruits. Although consumption of purple tomatoes would increase exposure to anthocyanins, our conservative estimates suggest that it would be no more than double for people currently consuming anthocyanin-colored foods. People who do not currently consume such foods would be exposed, on average, to approximately the same amount of anthocyanins as the colored food consumers.

### 9 Conclusions of Consultation Dossier

We concluded that the risk of detrimental effects on health parameters resulting from consuming purple tomatoes was extremely low because the purple tomatoes are substantially equivalent to other tomatoes except in the ways that they were intended to be different (i.e., increased levels of anthocyanins). The levels of anthocyanins are not raised to levels which pose a danger to health. The purple tomatoes do not contain proteins which might be regarded as allergenic or toxic, and the new proteins are rapidly digested by pepsin and consequently would be broken down in the GI tract, and are present at undetectable levels (<0.5 ng mL^−1^) in processed products from the purple tomatoes.

## Varietal Improvement

**Figure 5:**
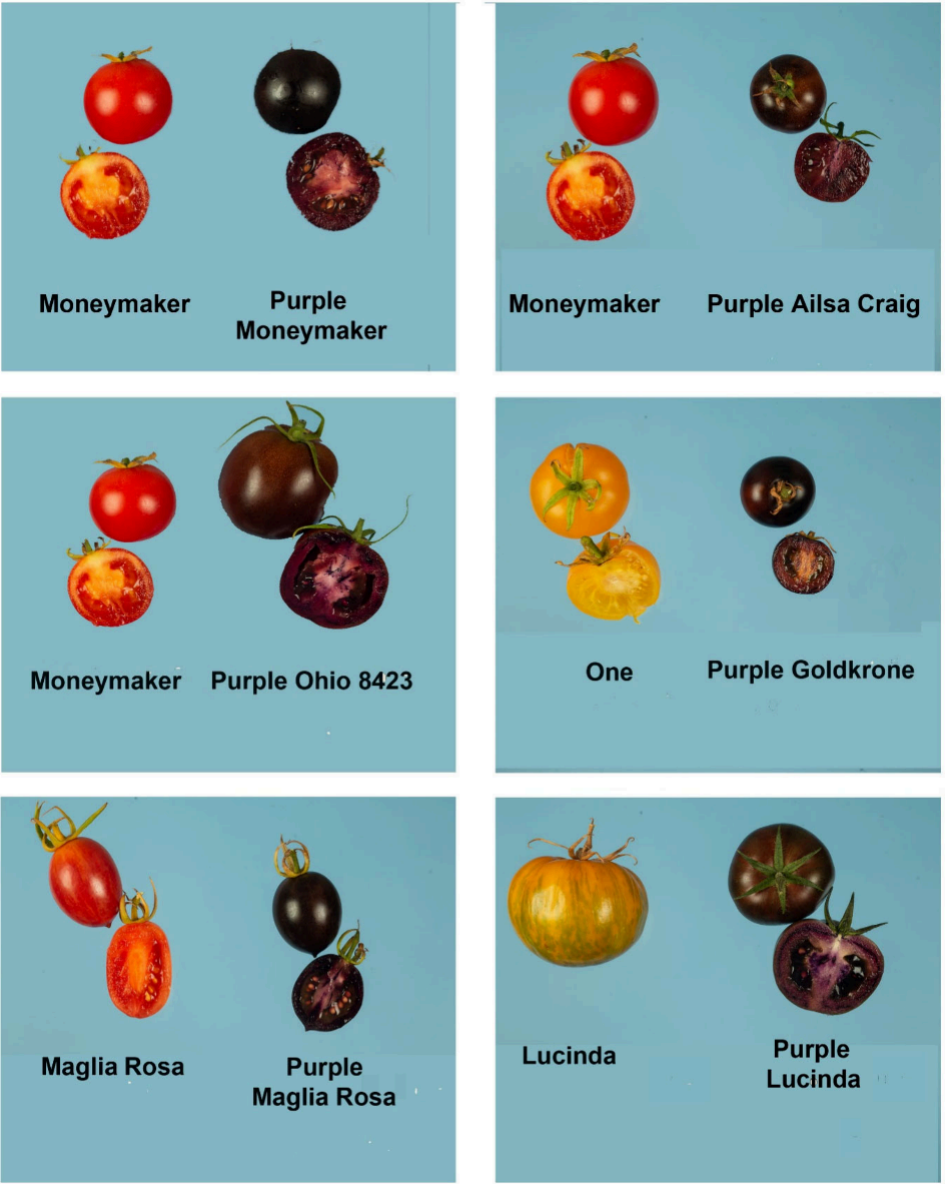
Purple fruit of different varieties. Fruit were photographed in the F2 generation from crosses between purple tomato in the Moneymaker background and Ailsa Craig, Ohio 8423, Goldkrone, Maglia Rosa, and Lucinda.

**Figure 6:**
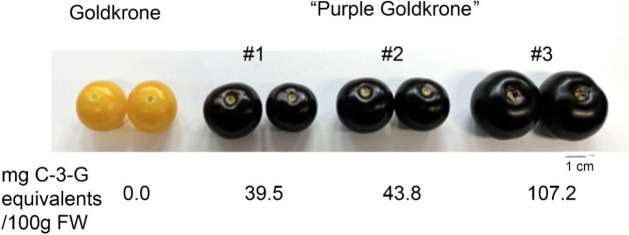
Purple fruit in the Goldkrone genetic background of tomato. Fruit from different lines segregating in the F3 from a cross between purple tomato in the Moneymaker genetic background and the yellow tomato variety Goldkrone. Lines producing smaller fruit were selected for home growers, those producing larger fruit were selected for sales as “snacking tomatoes” and “limited edition” products for supermarkets. The anthocyanin content of the different lines is shown below in mg cyanidin-3-glucoside (C-3-G) equivalents per 100 g fresh weight of fruit.

While FDA were considering our BNF application, we invested considerable effort in introgressing the purple tomato event into genetic backgrounds that would show off the anthocyanin content to best effect and enhance the taste/flavor profile of fresh tomatoes. This was not a trivial objective given the challenges of launching a GM product–the tomatoes had to look and taste great to stand a chance as a biotech food product. We crossed the purple tomato event in the MoneyMaker genetic background into a range of tasty heirloom varieties that were available without IP restrictions. The products of some of these crosses are shown in Figure 5. We selected indeterminate lines that flowered and fruited early, gave good yields, looked, and tasted great. The purple lines resulting from crosses to yellow tomato varieties (One and Goldkrone) were particularly promising. These yellow tomato varieties lack lycopene because they carry mutations in the *Red* (*R*) gene required for lycopene production in fruit (encoding phytoene synthase 1; PSY1). They are yellow because they accumulate chalcones and flavonols, particularly in their peel. These flavonoids can copigment the anthocyanins where they are present in the same cells, meaning that the purple trait is darker and bluer, a blue-ness enhanced by the lack of lycopene. Purple fruit (in the yellow fruit genetic background) were also noticeably sweeter. With a good yield of smaller, sweeter tomatoes we opted to take forward introgressions of the purple tomato event in the Goldkrone genetic background for proposed sales of seed of inbred lines to home growers (Figure 6).

## USDA/Animal and Plant Health Inspection Service (APHIS) Oversight

**Figure 7:**
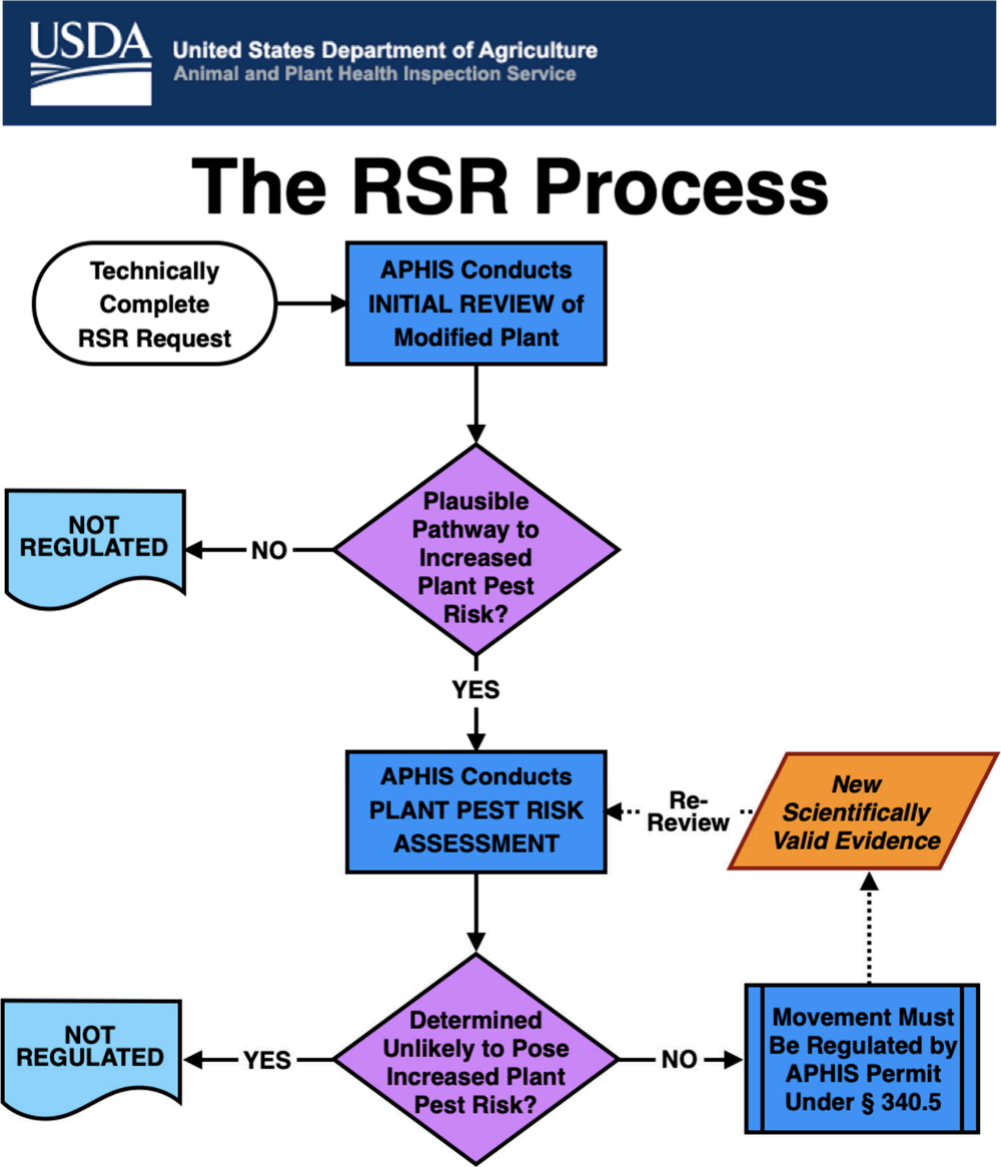
Workflow for RSR review of biotechnology traits in crops (courtesy of USDA/APHIS).

While we were awaiting FDA’s opinion on the safety of the purple tomato trait, the law governing regulatory approval of biotechnological traits in crops in the USA (the SECURE rule) underwent revision. The Revised Biotechnology Regulations focus on assessing an organism’s properties and not on the method used to produce it, enabling APHIS to regulate modified organisms with greater precision and reducing the regulatory burden for developers. After April fifth 2021, developers had the option to request a Regulatory Status Review (RSR) of a plant developed using genetic engineering that had not previously been evaluated and determined to be nonregulated. This process replaced the petition process in the preexisting regulations. We compiled an RSR application based on the data assembled for the notification of FDA. The RSR is a two step process with work flow illustrated in Figure 7. The initial review of whether the plant poses any plausible pathways to plant pest risk is rapid and aims to rule within 180 days of receipt of the application.

APHIS reviewed the modified purple tomato to determine whether there was a plausible pathway by which the tomato, or any sexually compatible relatives, would pose an increased plant pest risk relative to the plant pest risk posed by an appropriate tomato comparator. Based on information provided by NPS, publicly available resources, and APHIS’ familiarity with tomato and knowledge of the trait, phenotype, and mechanism of action, APHIS considered the

- biology of a comparator tomato and its sexually compatible relatives;
- the trait and mechanism-of-action of the modification;
- the effect of the trait and mechanism-of-action on the
  distribution, density, or development of the modified plant and its sexually compatible relatives,
  production, creation, or enhancement of a plant pest or a reservoir for a plant pest,
  harm to nontarget organisms beneficial to agriculture, and
  weedy impacts of the modified plant and its sexually compatible relatives.

APHIS did not identify any plausible pathway by which the modified tomato, or any of its sexually compatible relatives, would pose an increased plant pest risk relative to a comparator tomato, and consequently, the purple tomato was not a plant pest or a plant that required regulation because it was capable of introducing or disseminating a plant pest. APHIS therefore concluded that it had no authority to regulate it under 7 CFR part 340. Accordingly, the purple tomato was not subject to the regulations under 7 CFR part 340 and did not need to undergo Plant Pest Risk Assessment (PPRA). This “deregulation” cleared the path for commercialization of the purple tomatoes.

## The Conclusion of FDA Consultation on the Safety of Purple Tomatoes for Human Consumption

The APHIS decision that purple tomatoes would be deregulated in the USA was issued in September 2022, the first trait reviewed under the revised regulations. This advance allowed NPS to consider sales of fresh fruit for the first time, extending the reach of the company beyond a juice product anticipated in the FDA Biotechnology Notification Application. This change in anticipated use extended the time FDA took to review the NPS application for notification, but in June 2023 the FDA did complete their Voluntary Premarket Consultation on the purple tomato trait (BNF178).

The focus of FDA’s evaluation was on whether human food from the purple tomato event would contain new proteins or other substances that require premarket approval as food additives and whether human food from purple tomatoes is as safe as human food from other tomato varieties.

To assess these points, the FDA considered the origin of the two transcription factors used to enhance anthocyanin production in tomato fruit. The donor of the two genes was the garden snapdragon, *Antirrhinum majus*, which has been used in the human diet, in that the flowers are edible. The two transcription factors have a history of safe use and very similar transcription factors regulate anthocyanin production in many anthocyanin-rich fruit including aubergine and pepper as well as in wild species of the tomato family that produce anthocyanins in their fruit. Bioinformatic analysis showed no amino acid sequence similarity of Delila or Rosea1 to known allergens or toxins and the levels of the two proteins were below the limit of detection in the fruit (<0.5 ng Delila and <0.2 ng Rosea1 protein per mL juice). Both proteins were rapidly degraded by pepsin in simulated gastric fluid.

In assessing the safety of consuming tomatoes enriched in anthocyanins, FDA considered that there was a history of safe consumption, because the same anthocyanins are present in the skin of some purple-skinned tomato varieties and in eggplant and purple-fleshed potatoes. The levels in purple tomatoes, measured at 0.4 mg per g fresh weight in purple tomatoes (Moneymaker genetic background), would give an estimated dietary exposure of 100 mg anthocyanins/day at the mean, and 225 mg/day at the 90th percentile, exposures comparable to consuming other high anthocyanin foods.

FDA concluded that except for the intended anthocyanin change in purple tomatoes, human food from purple tomatoes was not materially different in composition, safety, and other relevant parameters from tomato-derived human food currently on the market, and consequently, use of genetically engineered purple tomatoes in human food did not raise issues that would require premarket review or approval by FDA.

Having been granted deregulatory status by USDA/APHIS and notified by FDA, we were in a position to move the purple tomatoes forward for sales in the USA. Sometime earlier, we had established a US subsidiary for this specific purpose. This company, Norfolk Healthy Produce, had been extremely active in promoting the purple tomatoes prior to regulatory approval through their Web site (https://www.norfolkhealthyproduce.com/), through disseminating articles such as ‘Learning to Love G.M.O.s’ published in the New York Times Magazine on July 20th, 2021 where Jennifer Kahn argued that overblown fears have turned the public against genetically modified food, but the potential benefits have never been greater. Kahn cited lead examples, including virus resistant papaya (which saved the Hawaiian papaya industry) and purple tomatoes. Norfolk Healthy Produce also arranged press interviews and press releases at each stage of the regulatory process. By July 2023 it was promoting local sales at farmers markets and fund raisers and showcased purple tomato and avocado crostini at the Ginko Bioworks FERMENT event. The purple tomatoes also received endorsements from celebrity chefs who were enthusiastic about their taste and exceptional appearance. In February 2024, purple tomatoes became the first genetically modified food crop to be marketed directly to home gardeners as seed. Previously, genetically modified foods were generally only available to commercial producers in the U.S. “We aim to show with this product and with this company that there’s a lot of benefits that can go to consumers through biotechnology, better taste, and better nutrition as prime examples,” said Nathan Pumplin, CEO of Norfolk Healthy Produce, a subsidiary of Norfolk Plant Sciences. Now the purple tomatoes are available in select groceries across the USA as Empress Limited Edition Tomatoes.

None of the progress made by NPS would have been possible without the dedication, help, and advice from scientific colleagues, friends, and advisors in the USA. These colleagues consistently supported us in our belief that biotechnology can produce products of benefit to consumers. Of course, the purple tomatoes are not yet available in Europe, despite the original funding being from the European Commission through the PROFOOD project. As scientists, we are hopeful that the regulatory assessments already undertaken in the USA will support approval in other countries. Of course, NPS is particularly interested in gaining approval for home growers in the UK. At the very least, we have laid a path to the market for a polyphenol-rich food product that could promote the health of consumers.
